# Exosomal circular RNAs: New player in breast cancer progression and therapeutic targets

**DOI:** 10.3389/fgene.2023.1126944

**Published:** 2023-02-28

**Authors:** Bashdar Mahmud Hussen, Sayran Mohamadtahr, Snur Rasool Abdullah, Hazha Jamal Hidayat, Mohammad Fatih Rasul, Goran Sedeeq Hama Faraj, Soudeh Ghafouri-Fard, Mohammad Taheri, Maryam Khayamzadeh, Elena Jamali

**Affiliations:** ^1^ Department of Medical Analysis, College of Pharmacy, Hawler Medical University, Erbil, Iraq; ^2^ Center of Research and Strategic Studies, Lebanese French University, Erbil, Iraq; ^3^ Medical Laboratory Science, Lebanese French University, Erbil, Iraq; ^4^ Department of Biology, College of Education, Salahaddin University-Erbil, Erbil, Iraq; ^5^ Department of Pharmaceutical Basic Science, Faculty of Pharmacy, Tishk International University, Erbil, Iraq; ^6^ Department of Medical Laboratory Science, Komar University of Science and Technology, Sulaimany, Iraq; ^7^ Department of Medical Genetics, School of Medicine, Shahid Beheshti University of Medical Sciences, Tehran, Iran; ^8^ Institute of Human Genetics, Jena University Hospital, Jena, Germany; ^9^ Urology and Nephrology Research Center, Shahid Beheshti University of Medical Sciences, Tehran, Iran; ^10^ Cancer Research Center, Shahid Beheshti University of Medical Sciences, Tehran, Iran; ^11^ Academy of Medical Sciences, Tehran, Iran; ^12^ Department of Pathology, Loghman Hakim Hospital, Shahid Beheshti University of Medical Sciences, Tehran, Iran

**Keywords:** breast cancer (BC), exosome, circular RNA (circRNA), exosomal circRNA (exo-circRNA), therapeutic target

## Abstract

Breast cancer is the most prevalent type of malignancy among women. Exosomes are extracellular vesicles of cell membrane origin that are released *via* exocytosis. Their cargo contains lipids, proteins, DNA, and different forms of RNA, including circular RNAs. Circular RNAs are new class of non-coding RNAs with a closed-loop shape involved in several types of cancer, including breast cancer. Exosomes contained a lot of circRNAs which are called exosomal circRNAs. By interfering with several biological pathways, exosomal circRNAs can have either a proliferative or suppressive role in cancer. The involvement of exosomal circRNAs in breast cancer has been studied with consideration to tumor development and progression as well as its effects on therapeutic resistance. However, its exact mechanism is still unclear, and there have not been available clinical implications of exo-circRNAs in breast cancer. Here, we highlight the role of exosomal circRNAs in breast cancer progression and to highlight the most recent development and potential of circRNAas therapeutic targets and diagnostics for breast cancer.

## 1 Introduction

Breast cancer (BC) is the most common cancer in women and represents a leading cause of death on a worldwide scale in women (Torre et al., 2015; Chen et al., 2016; Siegel et al., 2018). Breast cancer treatment options include surgery, radiotherapy, chemotherapy, endocrine therapy, and targeted therapy that have allowed for more accurate and tailored care for patients with early-stage. However, the 26% 5-year survival rate for metastatic BC patients shows that this treatment is still a real challenge (Malmgren et al., 2018). The exact molecular pathways behind its etiology are still unknown. Recent studies have shown a number of potential mechanisms by which non-coding RNAs (ncRNAs), like circRNAs, regulate the progression of BC.

Exosomes are nanometric vesicles of endosomal origin containing material from the host cell, such as proteins, lipids, DNA, and RNA (Hegmans et al., 2008). These vesicles are secreted by exocytosis and are taken up by other cells, influencing their functions and behavior, and can be used as a drug carrier for cancer therapy (Ghafouri-Fard et al., 2021a; Hussen et al., 2022). Tumorous cells use exosomes to communicate with their surroundings like other body cells. In recent years, research has shown that circRNA-enriched exosomes are involved in the hallmarks of cancer (Su et al., 2019). In addition, they have a vital role in promoting tumor progression by creating a suitable microenvironment for their proliferation and metastasis (Jia et al., 2017; Kalluri and LeBleu, 2020).

Recently, a new class of non-coding RNA called circular RNA (circRNA) was discovered in exosomes. These are single-stranded RNA molecules that have been covalently closed to make a loop (Jeck and Sharpless, 2014). Due to their distinctive structure, circRNAs have a longer half-life and better resistance capabilities to RNase and exonuclease than linear RNAs; and they are abundance in tumorous exosomes (Seimiya et al., 2020).

Initially, Li et al. were the first to report that exosomes contained a lot of circRNAs (Li et al., 2015a). They found that the entry of circRNAs into exosomes was controlled by a number of factors or pathways, such as changing the expression of key miRNAs in the parent cells. Another pathway through which circRNAs get access to exosomes is *via* binding to RNA-associated proteins (Bao et al., 2016). Exo-circRNAs can have an oncogenic role or suppressive effect by interfering with several biological pathways (Zhang et al., 2019) and sponging miRNAs which can inhibit gene expression at transcriptional level (Xing et al., 2020). More interestingly, new studies revealed that exo-circRNAs can contribute to cancer cell sensitivity to chemotherapy or hormonal and radiation therapy (Liu et al., 2021) (Yang et al., 2020) (Liang et al., 2019a).

Although our understanding of exo-circRNAs’ functions has greatly expanded during the past 2 decades, but their precise mechanism remains unclear. In addition, there is still no clinical implication of exo-circRNAs in BC. Therefore, the main aim of this review is to provide a comprehensive description of the different aspects of the exo-circRNAs that contribute to BCs’ development and to discuss the therapeutic targets based on exo-circRNA as anti-cancer responses.

## 2 Biogenesis of exosome

**FIGURE 1:**
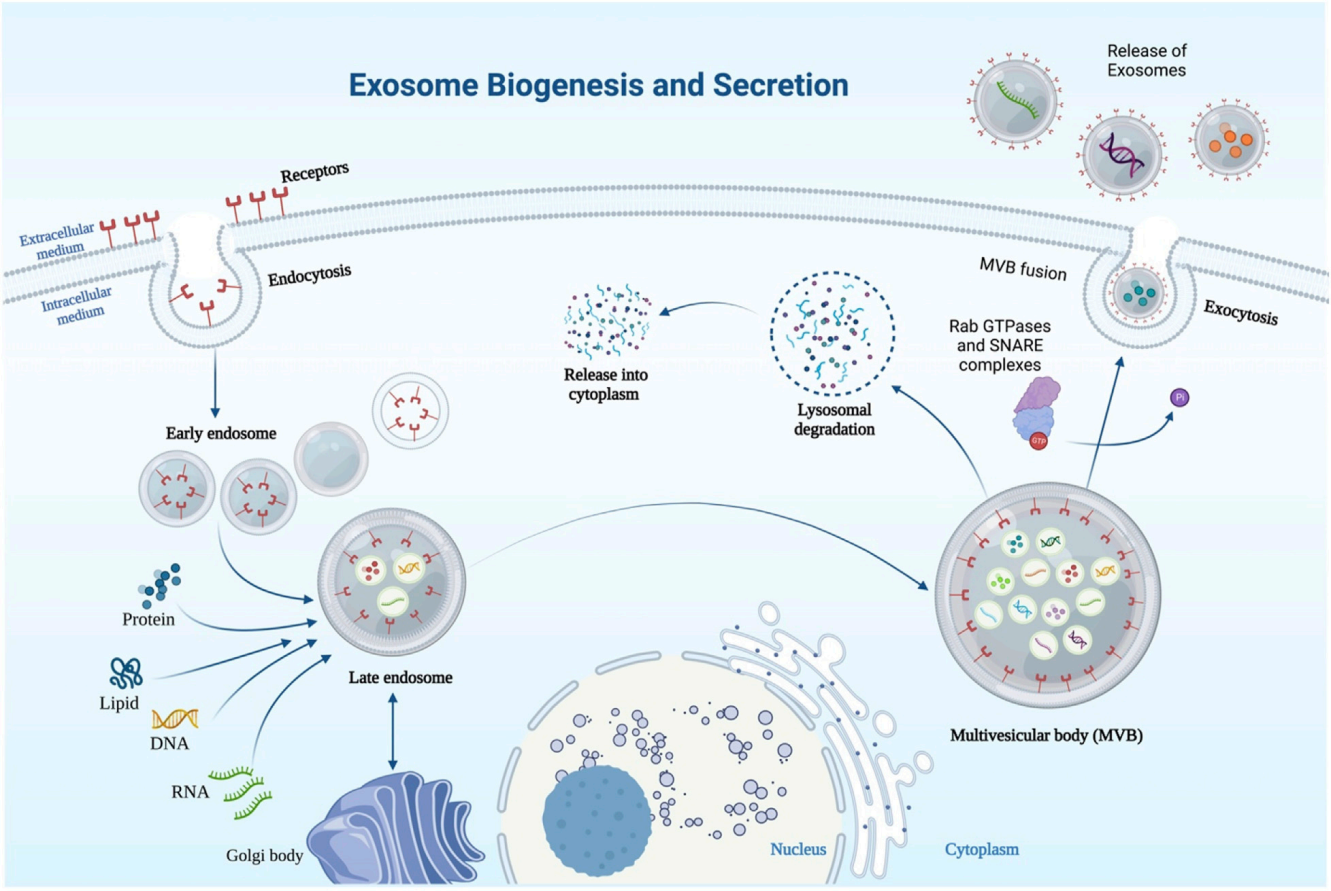
Schematic representation of exosome biosynthesis and secretion. Exosomes are formed through endocytic membrane invagination and ILV formation inside the cell. Payloads (RNA, proteins, DNA) are incorporated into ILV in an ESCRT-dependent or/and -independent manner during the maturation of early endosomes, which results in the formation of MVBs. MVBs with the plasma membrane results in the release of exosomes into the extracellular space, and MVBs can be transported along microtubules to the Golgi apparatus for endosome recycling or to lysosomes for degradation. Several key components including Rab GTPases and SNARE complexes are required for MVB fusion with the cellular membrane.

Endosomes serve as a starting point for the production of exosomes (Figure 1). The creation of intraluminal vesicles begins with the invagination of the plasma membrane, which leads to the production of early endosomes. Meanwhile, early endosomes develop into multivesicular bodies (MVBs) that store many intraluminal vesicles (van Niel et al., 2011; Raposo and Stoorvogel, 2013). Exosomes originate from these intraluminal vesicles. When MVBs connect with a cell membrane, they release exosomes into the surrounding environment.

The endosomal sorting complex necessary for transport (ESCRT) is a key player in exosome formation (Mattissek and Teis, 2014). ESCRT is made up of five protein complexes: ESCRT-0, ESCRT-1, ESCRT-2, ESCRT-3, and VPS4A (Henne et al., 2013). The clustering of ubiquitinated payloads is facilitated by ESCRT-0, which is composed of the substrate and signal-transducing adaptor protein for the tyrosine kinase. VPS4A disassembles the ESCRT machinery for recycling; ESCRT-I and ESCRT-II are responsible for bud formation, while ESCRT-III initiates vesicle scission (Hurley and Hanson, 2010). Both early endosomes and MVBs survive after critical subunits of the four ESCRTs are knocked out, despite potential substantial changes in the shape of the components of the endocytic pathway (Stuffers et al., 2009). It is possible that proteins other than ESCRT are involved in regulating endosomal sorting, such as CD63 (van Niel et al., 2011), CD81 tetraspanin ligands (Perez-Hernandez et al., 2013), CD9, CD82 (Chairoungdua et al., 2010), and RAB31 (Wei et al., 2021).

### 2.1 Secretion of exosomes

Multivesicular endosomes (MVEs) in cells can merge with the lysosome, sending their contents to be degraded, or they can fuse with the plasma membrane to send their contents out into the extracellular environment as exosomes (van Niel et al., 2011; Kowal et al., 2014). Secretion of exosomes is thought to be controlled by certain Rab GTPases (Stenmark, 2009). For instance, Rab27a, Rab27b (Ostrowski et al., 2010), and Rab35 (Yang et al., 2019a) are involved in MVE docking at the plasma membrane. If Rab27a or/and Rab27b silenced, exosome release is suppressed without noticeable changes to the exosomes’ protein composition or morphology. Additionally, two of Rab27’s effectors, Slp4 and Slac2b, are responsible for a decrease in exosome secretion upon their silencing (Ostrowski et al., 2010). Likewise, by preventing Rab27a from being degraded by the proteasome, KIBRA regulates exosome secretion (Song et al., 2019). Furthermore, vesicle-membrane SNAREs (v-SNAREs) and target-membrane SNAREs (t-SNAREs) worked together to control MVE fusion with the plasma membrane (Jahn and Scheller, 2006).

### 2.2 Biological characteristics of exosomes

Exosomes are made by almost every cell (Zhao et al., 2021). They are detected to be circulating in fluids and secretions of the body, like urine, blood, bile, tears, semen, saliva, cerebrospinal and amniotic fluids (Geng et al., 2020). Moreover, exosomes play crucial regulatory roles in intercellular communications, locally and systematically (Wang et al., 2019a) by acting as signalling vesicles in autocrine, endocrine, juxtracrine, and paracrine (EL Andaloussi et al., 2013). Moreover, they can transport cargo from cell to cell, causing phenotypic alterations in recipient cells (Mathieu et al., 2019).

Exosomes have been implicated in many biological processes and show an essential role in pathological and physiological situations, including cancer, circulatory and metabolic abnormalities, immune responses, and inflammatory illnesses (Wang et al., 2019a; Kalluri and LeBleu, 2020). For example, through releasing cytokines and other bioactive molecules, exosomes create an appropriate microenvironment for tumor growth and regulate cell reproduction, metastasis, and drug resistance (Ye et al., 2022). Exosomes also contribute to early tumorigenesis in BC, can carry signalling molecules to cancer cells inside the TME, assist cancer cells in evading an immune response, promote angiogenesis, and alter the tumor microenvironment among other functions (Jia et al., 2017).

## 3 Biogenesis of circRNAs

**FIGURE 2:**
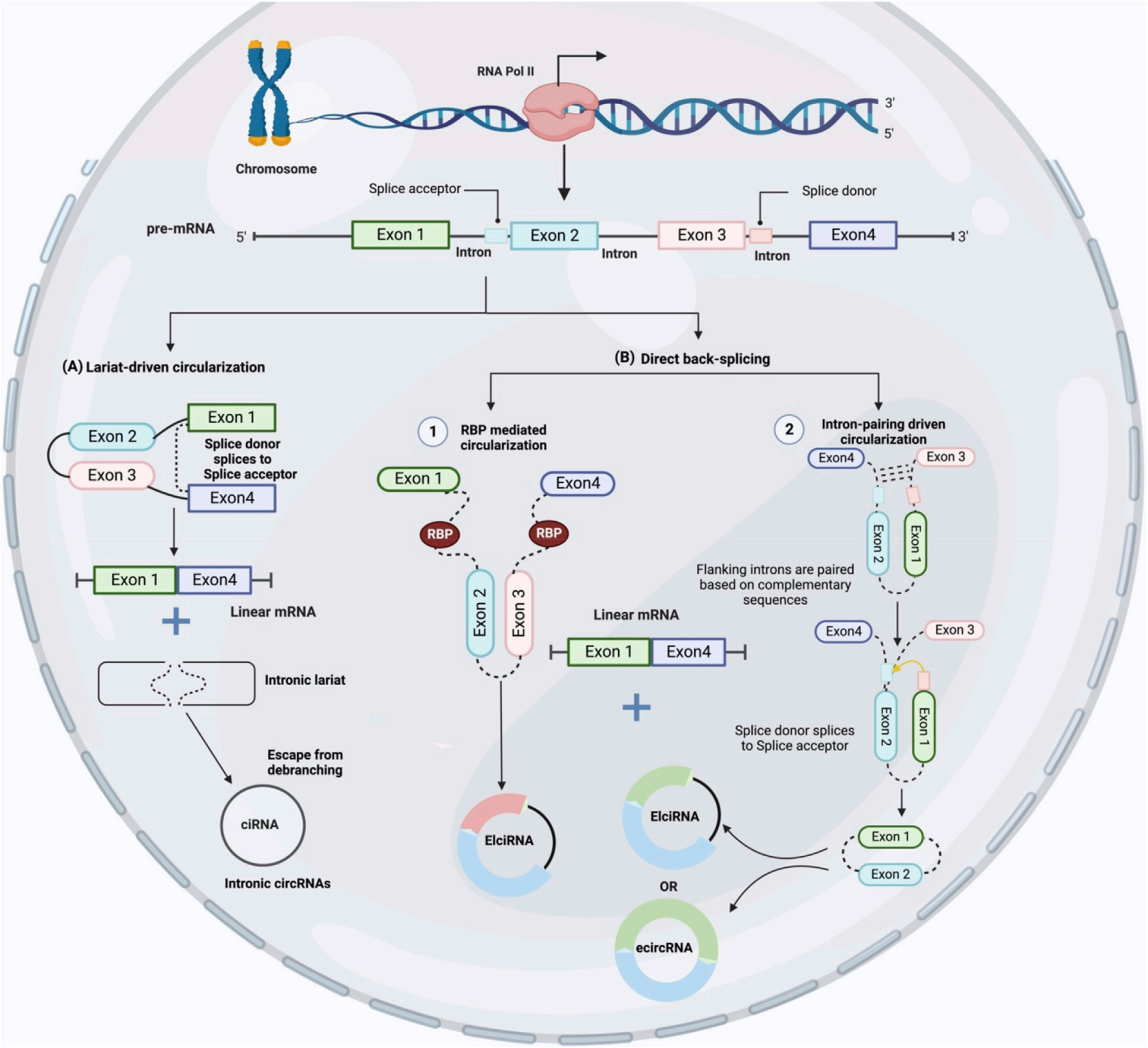
CircRNA biogenesis. **(A)** Exon skipping creates an mRNA with exons one and four and a lariat structure or intronic lariat escape from debranching and produced intronic circRNAs (ciRNAs). **(B)** Back-splicing occurs initially, followed by further processing to create linear mRNA from circRNA with exons and introns. The first type, called “intron-pairing driven circularization,” occurs when complementary segments on either side of an intron pair up to carry the splice sites closer together and facilitate circularization. The second type, called “RBP mediated circularization,” occurs when RBPs bind with the intron binding sites to bring the splice sites closer together and initiate circularization.

CircRNAs are synthesized by RNA polymerase II transcription from pre-mRNA back-splicing, which links a 5′ splice site (downstream splice donor site) to a 3′ splice site (upstream acceptor splice site) (Ashwal-Fluss et al., 2014). Through alternative back-splicing, multiple circRNAs can be created from identical sequences. Despite decades of research, the precise mechanism underlying circRNA production is not fully understood. CircRNAs are categorized into three classes based on their structure and cycling mechanisms: exonic circRNA (ecircRNA) (Zhang et al., 2014; Chen et al., 2015), circular intronic RNA (ciRNA) (Zhang et al., 2013), and exon-intron circRNA (ElciRNA) (Li et al., 2015b). EcircRNAs are primarily found in the cytoplasm and have one or more exons resulting from alternative splicing (Zhang et al., 2014). The biosynthesis of ecircRNAs can be explained by one of three potential models: lariat-driven, RBP-mediated, and intron-pairing-driven (Jeck et al., 2013). When RNA is folded, exons are skipped and ecircRNAs are created. These rearrangements cause the creation of lariat structures, which are clusters of nearby exons and introns previously located far from each other. After the intron sequence is spliced out using the lariat structure, circular RNAs are produced (Figure 2). It is believed that back-splicing and canonical splicing are linked because the majority of highly expressed circRNAs are constructed from many internal exons of pre-mRNAs and a small number of introns (Zhang et al., 2014). Nevertheless, identifying the co-expression of circRNAs and their putative linear RNAs with exon exclusion has been challenging because of the quick degradation of untranslated linear RNAs (Jeck and Sharpless, 2014).

CircRNA biogenesis could be divided into two subprocesses based on the sequence of their basic steps. Lariat-driven circularization or back-splicing of a linear RNA including skipped exons and a long lariat of introns and exons generates a circRNA using the canonical splicing pathway which is called exon skipping model (Figure 1A). However, If the RNA precursor undergoes back-splicing early, a circRNA and an intermediate containing introns and exons will be generated. After this, a linear RNA is synthesized from the RNA precursor which is called direct back splicing (Figure 1B). Recent studies have shown that both developed models are successful *in vivo* (Lasda and Parker, 2014; Chen and Yang, 2015). Direct back splicing is further categorized into two other groups: “RBP mediated circularization” and “intron-pairing driven circularization” based on the differences in circularization mechanisms (Huang and Zhu, 2021). Furthermore, it is exciting to discover how these factors control circRNA expression and how they modify during carcinogenesis.

### 3.1 Biological characteristics of circular RNAs

Expression and regulation of circRNAs take place specifically by cells and tissues at different developmental stages (Conn et al., 2015). The majority primarily exists in the cytoplasm. Scientists have studied their function as post-transcriptional regulators (Han et al., 2018). Although there is no comprehensive description of circRNAs properties and biological implications (Tran et al., 2020), thus far, it has been shown to act like miRNA sponges, interact with proteins, regulate the splicing or transcription of genes, translate proteins, and exert epigenetic regulations (Han et al., 2018). Having diverse functions in the body, circRNAs involvement in various body diseases, such as cardiovascular diseases (Altesha et al., 2019), renal diseases (Jin et al., 2020a), chronic liver diseases (Zeng et al., 2021), skin diseases (Wu et al., 2020) and cancers (Ghafouri-Fard et al., 2022a; Ghafouri-Fard et al., 2022b).

Studies continuously demonstrate that abnormally expressed circRNAs are associated with several features of cancer (Li J. et al., 2020), in particular carcinogenesis, programmed cell death, proliferation, invasiveness, metastatic abilities, resistance to radiotherapy and chemotherapy, and cancer prognosis (Geng et al., 2020).

Dysregulation in circRNA gene expression is considered one of the important factors causing the onset and progression of gynecological cancers (Tran et al., 2020). In addition, numerous studies over the last years have shown circRNAs to be tightly linked to BC, and these studies can be categorized into two major groups: those that investigate the regulatory effect in BC development and those focused on detecting different patterns of expression to elucidate possible biomarkers for diagnosis of BC or molecular subtypes (Ghafouri-Fard et al., 2021b).

## 4 Biological implications of exosomal circRNAs in breast cancer

### 4.1 Tumor regulation, cell proliferation, and angiogenesis

Exo-circRNAs’ contribution to tumor regulation, proliferation, and angiogenesis has been shown in many studies. When Li et al. studied tumor and paracancerous sample tissues of BC patients, they detected significant overexpression of circ_002178 in cancer tissue compared to surrounding cells. They also found that overexpression of circ_002178 *in vitro* caused a considerable increase in the rate of survival and the number of SUM149PT cell clones, which facilitates tumorigenesis through targeting miR-1258 and modulating the expression of KDM7A (Li W. et al., 2020). Further, Liu et al. used the gain and loss of function approach to study the functional role of hsa_circRNA_002178 in BC angiogenesis. They revealed that overexpression of has_circRNA_002178 was associated with poor prognosis in their microarray-based gene expression analysis. Later they proved that hsa_circRNA_002178 knockdown inhibited angiogenesis by decreasing cell survival, energy usage, and the ability to form tubes (Liu et al., 2020a). Likewise, Cai et al. used the same gain and loss of function to study hsa_circ_0000515. First, they detected overexpressed hsa_circ_0000515 in BC tissues. Afterward, they silenced hsa_circ_0000515 in the MCF-7 cell line. Finally, they found that MCF-7 cells were neither able to progress through their cell cycle nor able to proliferate or invade, and their angiogenetic potential was diminished through sponging miR-296-5p and modulating CXCL10 expression (Cai et al., 2021).

Furthermore, Liang and his team performed a circRNA microarray to screen for dysregulated circRNA in BC tissues. Among 2,587 abnormally expressed circRNA, there was significant overexpression of circ-ABCB10 with five to ten times more in BC cells than normal ones. Then, their bioinformatics study found that miR-1271 could represent a target for circ-ABCB10. They also found that knocking down circ-ABCB10 led to cell cycle arrest in the G0/G1 phase, suppression of colony formation, and inhibition of cell proliferation *in vitro* (Liang et al., 2017). Circ-Dnmt1 (hsa_circRNA_102,439) was overly expressed in BC cells, which caused inducing of proliferation and survival of cells. It also led to stimulation of cellular autophagy, inhibition of cellular biological aging, and increment of tumor growth (Du et al., 2018). These outcomes resulted from circ-Dnmt1 binding to p53 and Auf1, which triggered nuclear translocation of these tumor suppressor proteins and lowered p53 transcription (Tesfaye et al., 2021). Table 1 shows the expression levels of several different carcinogenic exo-circRNAs in BC along with their targets and hallmarks.

**TABLE 1:** Overexpressed exo-circRNAs that act as an oncogene in breast cancer.

Circular RNA	Target	Animal model	Cell line	Type of specimen	Hallmark	Association with clinical characteristics and outcome	Ref.
circ_002178	miR-328-3p, COL1A1	Nude mice	MCF-10A, MDA-MB-231, MCF-7, T47D, BT549, HUVECs	Tumour and adjacent normal tissues from 70 patients	Angiogenesis (+)	Prognosis	Liu et al. (2020a)
					Proliferation (+)		
	miR-1258, KDM7A	Mice	MDA-MB-231, SUM159PT, MDA-MB-468, HCC 1806, SUM149PT	Tumour and adjacent normal tissues from 83 patients	Tumourigenesis (+)	Prognosis	Li W. et al. (2020)
					Invasion (+)	TNM	
					Migration (+)	Lymph node metastasis	
						Tumor size	
circ-DNMT1	p53, Auf1	Nude mice	293T, HUVEC, HaCaT, BEAS2B Jurkat, MCF10A, MCF-7, MDA-MB468, SK-BR-3, HTB126, MDA-MB-231, PC3, HepG2,H460, JHH1, Hela, Du145, LnCap, SNU449	Tumour and adjacent normal tissues	Proliferation (+)	N/A	Du et al. (2018)
hsa_circ_0008039	miR-515-5p, CBX4	Nude mice	MCF-7, SKBR3, MCF10A	Tumour and adjacent normal tissues from 35 patients	Proliferation (+)	N/A	Huang et al. (2020)
					Migration (+)		
					Invasion (+)		
circRNF20	miR-487a, HIF-1α, HK2	Nude mice	MCF-10A, MDA-MB-453, MCF-7, MDA-MB-231, MDA-MB-468	Tumour and adjacent normal tissues from 50 patients	Tumourigenesis (+)	Lymph node metastasis	Cao et al. (2020)
					Proliferation (+)	Tumor size	
					Apoptosis (-)	Prognosis	
circ_0007255	miR-335-5p, SIX2	Nude mice	MCF-10A, MCF-7, MB468, MB231, T47D	Tumour and adjacent normal tissues from 50 patients, serum from 50 patients and 48 healthy volunteers	Migration (+)	Prognosis	Jia et al. (2020)
					Invasion (+)		
circABCC4	miR-154-5p, NF-κB and Wnt/β-catenin signal pathway	N/A	MDA-MB-231, MCF-7	Tumour and adjacent normal tissues from 25 patients	Migration (+)	N/A	Jiang and Cheng (2020)
					Invasion (+)		
					Apoptosis (-)		
circMYO9B	miR-4316, FOXP4	Nude mice	MCF-10A, MDA- MB-453, BT474, T47D, MCF-7, MDA-MB231	41 cancer tissues with 21 normal adjacent tissues	Proliferation (+)	Prognosis	Wang et al. (2018b)
					Migration (+)	Lymph node metastasis	
					Invasion (+)	TNM stage	
						Tumor size	
hsa_circ_001783	miR-200c-3p, ETS1, ZEB1, ZEBI2	N/A	MCF-10A, MCF-7, 47D, MDA-MB231, SK-BR-3, BT474, MAD-MB-468,	Tumour tissues of 136 breast cancer patients, 18 paired normal tissues	Proliferation (+)	Tumour size Lymph node metastasis	Liu et al. (2019)
					Migration (+)	TNM stage	
					Invasion (+)	Prognosis	
circ‐TFF1	miR‐326, TFF1	Nude mice	MCF‐10A, BT‐549, MCF‐7, MDA-MB‐231, MDA‐MB‐453	Healthy breast tissues, BC tumor, and adjacent normal tissues	Proliferation (+)	N/A	Pan et al. (2020)
					Migration (+)		
					Invasion (+)		
					EMT (+)		
hsa_circ_0131,242	hsa-miR-2682	N/A	MCF-10A, T47D, BT549, MDA- MB-468, MDA-MB -231, HCC1806	Tumour and adjacent normal tissues from 120 patients	Proliferation (+)	Tumor size	Li Y. et al. (2020)
					Migration (+)	TNM stage Prognosis	
circKIF4A	miR-152, ZEB1	N/A	MCF-10A, MDA-MB-231, MCF-7	Tumour and normal tissues from 41 patients	Migration (+)	Tumor size	Jin et al. (2020b)
					Invasion (+)	TNM stage	
					Apoptosis (-)		
	miR-375, KIF4A	Nude mice	MCF10A, SKBR3, BT474, MCF-7, T47D, BT549, MDA-MB-468, MDA-MB-453, HCC38, MDA-MB-231	N/A	Proliferation (+)	Lymph node metastasis Tumour size TNM stage	Tang et al. (2019)
					Migration (+)		
circUBE2D2	miR-512-3p, CDCA3	Nude mice	BT-549, SUM-159, MDA-MB-231, MDA-MB-468, HCC38, MCF-10A	Tumour and adjacent surrounding tissues from 66 patients	Proliferation (+)	TNM stage	Dou et al. (2020)
					Migration (+)	Lymph node metastasis	
					Invasion (+)	Prognosis	
					Apoptosis (-)		
circHMCU	let-7 miRNAs, MYC, HMGA2, CCND1	Nude mice	MDA-MB-468, MDA-MB-231, MCF7	267 BC tissues, 58 normal BC tissues	Proliferation (+)	Lymph node metastasis	Song et al. (2020)
					Migration (+)	Node stage Histological grade	
					Invasion (+)	Tumour and node stage	
					Apoptosis (-)	Prognosis	
cirCHIPK3	miR-193a, HMGB1, PI3K/AKT axis	Nude mice	MCF-10A, MDA-MB-468c, MCF-7, MDA-MB-453, MDA-MB-231	Tumour and adjacent normal tissues from 50 patients	Proliferation (+)	Prognosis	Chen et al. (2020)
					Migration (+)	Tumor size	
					Invasion (+)	TNM stage	
						Lymph node metastasis	
circ_0041732	miR-149-5p, FGF5	Nude mice	MDA-MB-231, BT-549, HUVEC, MCF-10A	Tumour and adjacent normal tissues from 57 patients	Proliferation (+)	N/A	Li et al. (2022a)
					Migration (+)		
					Invasion (+)		
					Apoptosis (-)		
hsa_circ_0103,552	miR-515-5p, CYR61	N/A	MCF10A, ZR-75-1, MCF7, Bcap-37, MDA-MB-231, HCC1937	Tumour and adjacent normal tissues from 42 patients	Proliferation (+)	Tumor size	Huang et al. (2021)
					Migration (+)	Lymphatic metastasis	
					Invasion (+)	Prognosis	
hsa_circ_0011946	miR26a/b, RFC3	N/A	HS-578T, SKBR-3, T47D, BT549, MCF-7, MDA-MB-231	Tumour and adjacent normal tissues from 3 patients	Migration (+)	N/A	Zhou et al. (2018)
					Invasion (+)		
circANKS1B	miR-152-3p, miR-148a, USF1, TGF-β1	Nude mice	MCF10A, BT549, T47D, MCF-7, MDA-MB-468, SK-BR-3, MDA-MB-231	23 fresh frozen tumors and surrounding normal tissues, 165 FFPE tumour tissues, 40 normal tissues	Migration (+)	Lymph node metastasis	Zeng et al. (2018)
					Invasion (+)	Prognosis	
					EMT (+)		
circAGFG1	miR195-5p, CCNE1	Nude mice	MCF-10A, MDA-MB-231, BT-549, MDA-MB-453, SUM-159, MDA-MB-468,	Tumour and adjacent normal tissues from 40 patients	Tumorigenesis (+)	Clinical stage Prognosis	Yang et al. (2019c)
					Angiogenesis (+)		
					Proliferation (+)		
					Migration (+)		
					Invasion (+)		
					Apoptosis (-)		
circ_0005230	miR-618, CBX8	Nude mice	MCF10A, MDA-MB-231, BT-20, MCF7, SKBR3, MDA-MB-436, T47D	Tumour and adjacent normal tissues from 76 patients	Proliferation (+)	Tumour size	Xu et al. (2018)
					Migration (+)	TNM stage	
					Invasion (+)	Lymph node metastasis	
					Apoptosis (-)		
circIFI30	miR520b-3p, CD44	Nude mice	MCF-10A, BT-549, MDA-MB-468, DA-MB-231	Tumour and adjacent normal tissues from 38 patients	Tumorigenesis (+)	TNM stage Prognosis	Xing et al. (2020)
					Proliferation (+)		
					Migration (+)		
					Invasion (+)		
					Apoptosis (-)		
					EMT (+)		
hsa_circ_0007534	miR-593, MUC19	N/A	MCF-10A, MDA-MB-453, MDA-MB-231, SKBR-3, MDA-MB-468, MCF-1	Tumour and adjacent normal tissues from 40 patients	Proliferation (+)	Prognosis	Song and Xiao (2018)
					Invasion (+)		
					Apoptosis (-)		
circDENND4C	miR-200c, miR-200 b	Nude mice	SK-BR-3, MDA-MB-453, MCF-10A	Tumour and surrounding normal tissues from 43 patients	Migration (+)	Lymph node metastasis TNM stage	Ren et al. (2019)
					Invasion (+)	Tumour size	
circPLK1	miR-296-5p, PLK1	Nude mice	MCF10A, MDAMB-468, BT549, MDA-MB-453, HCC38, MDA-MB-231	Tumour and surrounding normal tissues from 57 patients	Proliferation (+)	Tumour size	Kong et al. (2019)
					Invasion (+)	TNM stage	
						Lymph node metastasis	
circEPSTI1	miR-4753, miR-6809, BCL11A	Nude mice	HCC38, MCF-10A,184A1, MCF-7, BT549, HCC 1806, MDA-MB-231, Skbr-3, MDA-MB-415, MDA-MB-468, T47D, BT474, HEK 293T	Tumour and adjacent normal tissues from 240 patients	Proliferation (+)	Lymph node metastasis	Chen B. et al. (2018)
					Apoptosis (-)	Tumour size Prognosis	
						TNM stage	
hsa_circ_0000515	miR-296-5p, CXCL10	Nude mice	MCF10A, MCF-7, MDA-MB-231, MDA-MB-157, SK-BR-3, SUM-159	Tumour and adjacent normal tissues from 340 patients	Angiogenesis (+)	Prognosis	Cai et al. (2021)
					Proliferation (+)		
					Invasion (+)		
					Apoptosis (-)		
circCDYL	miR-1275, ATG7, ULK1	Nude mice	MCF-7, MDA-MB-231	Plasma from 30 early BC patients, 14 benign patients, and 18 metastatic BC patients, tumor and adjacent normal tissues from 113 patients,	Proliferation (+)	Tumor size	Liang et al. (2020)
						Lymph node metastasis	
						Prognosis	
						Response to therapy	
circGFRA1	miR-34a, GFRA1	Nude mice	184A1, MCF10A, BT474, BT549, MCF-7, MDA-MB-231, BT-483, BT-20, T47D, MDA-MB-468, SKBR3	Tumour and adjacent normal tissues from 222 patients	Proliferation (+)	Tumor size	He et al. (2017)
					Apoptosis (-)	TNM stage	
						Lymph node metastasis	
						Prognosis Histological grade	
hsa_circ_0004771	miR-653, ZEB2	Nude mice	MCF-10A, BT549, T47D, MCF-7, MDA-MB-231, Hs-578T	Tumour and adjacent normal tissues from 51 patients	Proliferation (+)	Prognosis	Xie et al. (2019)
					Apoptosis (-)		
circSEPT9	E2F1, EIF4A3, miR-637, LIF/Stat3 signalling pathway	Nude mice	SUM-159, BT-549, MDA-MB-453, MDA-MB-231, MDA-MB-468, MCF-10A	Tumour and adjacent normal tissues from 60 patients	Proliferation (+)	Lymph node metastasis Prognosis TNM stage	Zheng et al. (2020)
					Migration (+)		
					Invasion (+)		
					Apoptosis (-)		
FECR1 circular RNA	DNMT1, TET1	N/A	ZR751, MDA-MB231, T47D, MCF7, SKBR3, BT474, 293 T	Tumour and adjacent normal tissues	Proliferation (+)	N/A	Chen N. et al. (2018)
					Invasion (+)		
					Apoptosis (+)		
circIRAK3	miR-3607, FOXC1	Nude mice	MCF10A, HEK-293T T47D, SK-BR-3, BT-549MCF7, MDA-MB-231, HCC 1937, BT-474, HCC 1806, MDA-MB-157, HCC70, MDA-MB-436	Tumour and adjacent normal tissues from 35 patients	Migration (+)	Recurrence Prognosis	Wu et al. (2018)
					Invasion (+)		
circ_0006528	miR-7-5p, Raf1, MAPK/ERK signalling pathway, MEK1/2, ERK1/2	N/A	MDA-MB-231, BT-549, T-47D ZR-75-30, Hs578T, MCF-7, BT-474	97 tumour and 29 adjacent normal tissues from BC patients	Tumorigenesis (+)	Prognosis TNM stage	Gao et al. (2019)
					Proliferation (+)		
					Migration (+)		
					Invasion (+)		
					Apoptosis (-)		
hsa_circ_0052112	miR-125a-5p, ZNF83	N/A	MDA-MB-231, MCF-7	N/A	Migration (+)	Prognosis	Zhang et al. (2018b)
					Invasion (+)		
circFBXL5	miR-216b, HMGA2	Nude mice	MDAMB-231, MDA-MB-453, MCF-10A	39 tumor and adjacent normal tissues	Migration (+)	Tumor size	Zhu et al. (2021)
					Invasion (+)	TNM stage	
					Apoptosis (-)		
	miR‐660, SRSF6	Nude mice	MCF-10A, T47D, SKBR3, BT474, HCC38, BT549, MDA‐MB‐453, MDA‐MB‐231, MDA-MB-468	Primary BC tissues and metastatic lung tissues, BC tissues from 150 patients	Proliferation (+)	Prognosis	Zhou et al. (2020a)
					Migration (+)		
circ-TFCP2L1	miR-7, PAK1	N/A	MCF-10A SUM1315, HCC 1937, MDA-MB231	Tumour and adjacent normal tissues from 39 patients	Proliferation (+)	TNM stage	Wang et al. (2019b)
					Migration (+)		
hsa_circ_0001982	miR-143	N/A	HBL-100, MDA-MB-435, MDA-MB-231, MDAMB-468, MCF-7	Tumour and adjacent normal tissues from 29 patients	Proliferation (+)	N/A	Tang et al. (2017)
					Migration (+)		
					Invasion (+)		
					Apoptosis (-)		
circ-UBAP2	miR-661, MTA1	Nude mice	MDA-MB-231, SK-BR3, MCF-10A, MDA-MB-468BT-20, MCF-7, T47D	Tumour and adjacent normal tissues from 78 patients	Proliferation (+)	Tumor size	Wang et al. (2018c)
					Migration (+)	TNM stage	
					Apoptosis (-)	Lymph node metastasis	
						Prognosis	
hsa_circ_0072995	miR-30c-2-3p	Nude mice	MDA-MB-231, MCF-7	N/A	Migration (+)	Prognosis	Zhang et al. (2018c)
					Invasion (+)		
circ_0001667	miR-4458, NCOA3	N/A	MDA-MB-231, MCF-7	Tumour and adjacent normal tissues from 61 patients	Proliferation (+)	N/A	Cui et al. (2022)
					Migration (+)		
					Invasion (+)		
circRAD18	miR-3164, miR-208a, FGF2, IGF1	Nude mice	HEK 293T, MCF-10A, BT549, MDA-MB-231, BT474, MDA-MB-468, Skbr-3, HCC38, MDA-MB-453, HCC 1806, MCF-7, T47D	Tumour and adjacent normal tissues from 126 patients	Proliferation (+)	Tumor size	Zou et al. (2019)
					Migration (+)	Clinical stage	
					Apoptosis (-)	Prognosis	
circACAP2	miR-29b-3p, miR-29a-3p, COL5A1	Nude mice	MCF-10A, MDA-468, MDA-MB-453, MDA-MB-231	53 samples of BC tumor and adjacent normal tissues	Proliferation (+)	N/A	Zhao et al. (2020a)
					Migration (+)		
					Invasion (+)		
					Apoptosis (-)		
circGNB1	miR141-5p, IGF1R	Nude mice	MCF-10A, HCC 1806, MDA-MB-231, MCF-7, BT549, BT474, HCC38, MDA-MB-361, T47D, SKBR-3	Tumour and adjacent normal tissues from 222 BC patients	Proliferation (+)	Tumour size Clinical stage	Liu et al. (2020c)
					Migration (+)	Prognosis	
hsa_circRPPH1_015	miR-326, ELK1	Nude mice	HBL-100, MDA-MB-231, MCF-7, BCAP, MDA-MB-435	Tumour and adjacent normal tissues from 86 patients	Proliferation (+)	Tumor size	Zhao et al. (2020b)
					Invasion (+)	Lymph node metastasis	
						Pathological grade	
						Clinical stage	
circ-Amotl1	c-myc	Nude mice	HepG2, MDA-MB-231	Tumour and adjacent normal tissues samples	Tumorigenesis (+)	N/A	Yang et al. (2017)
					Proliferation (+)		
					Invasion (+)		
					Apoptosis (-)		
circSKA3	Itgb1, Tks5	Nude mice	MCF10A, Jurkat, 293T, HaCaT, MCF-7, MDA-MB-468, MDA-MB-231, SK-BR-3, HTB126, HeLa, H460, CI3K, PC3, LnCap, A549, Du145, HepG2, SNU449	61 BC specimen and 55 benign tissues specimen	Proliferation (+)	Stage of BC	Du et al. (2020)
					Invasion (+)		
					Migration (+)		
hsa_circ_001569	PI3K-AKT pathway	N/A	MCF-10A, MDA-MB-453, MCF-7, MDA-MB-231, MDA-MB-468	61 BC samples and 55 benign tissues	Proliferation (+)	Lymph node metastasis Clinical stage Prognosis	Xu et al. (2019a)
					Migration (+)		
					Invasion (+)		
					Apoptosis (-)		
circVAPA	miR-130a-5p	N/A	MDA-MB-231, MCF-7	Tumour and adjacent normal tissues from 29 patients	Proliferation (+)	N/A	Zhou et al. (2020b)
					Migration (+)		
					Invasion (+)		
					Apoptosis (-)		
circBCBM1	miR-125a, BRD4, MMP9, Sonic hedgehog (SHH) signalling pathway	Nude mice	231-BR, MDA-MB-231, T47D, BT-474	13 pairs of BC and adjacent normal tissues, 6 BC brain metastasis tissues, plasma samples from 20 BC and BCBM patients	Proliferation (+)	Prognosis	Fu et al. (2021)
					Migration (+)		
					Apoptosis (-)		
circRNA_069718	Wnt/β-catenin pathway genes (β-catenin, c-myc, and cyclin D1)	N/A	MCF-10A, MDA-MB-468, MCF-7, T47D, MDA-MB-231, BT20	35 tumor and adjacent normal tissues samples	Proliferation (+)	TNM stage	Zhang et al. (2019)
					Invasion (+)	Lymph node metastasis	
						Prognosis	

Cancer cells utilize a significant amount of their energy using anaerobic glycolysis (Warburg effect), a phenomenon of changing glucose to lactate for energy generation, succeeded by lactate fermentation in the absence of oxygen (Fadaka et al., 2017). In BC cells, circRNF20 promoted anaerobic glycolysis by increasing glucose uptake, lactate production, and ATP levels, in addition to reducing apoptosis and increasing proliferation (Cao et al., 2020).

Despite this, several circRNAs are extensively downregulated *in vitro* and *in vivo* studies of BC; these circRNAs inhibit tumor growth by sponging oncogenic miRNA or targeting other oncogenic products (Table 2). For example, Zhang and Ling Mao found circ_0000442 to be downregulated in cell lines and patient specimens. Using a variety of methods, they were able to identify the tumor suppressor circ_0000442 by its ability to sponge miR-148b-3p and decrease its proliferative effect. Furthermore, the G1 arrest effect was enhanced by circ_0000442 overexpression in cell lines, and colony formation was inhibited (Zhang and Mao, 2021). In the same way, Peng and Wen found that circDDX17 expression was low and there was a direct interaction between circDDX17 and miR-605, which controlled the expression of cell cycle genes (increased p21 expression and suppressed CDK1 expression) (Peng and Wen, 2020).

**TABLE 2:** Exo-circRNAs are under-expressed and act as tumor suppressors in breast cancer.

Circular RNA	Target	Animal model	Cell line	Type of specimen	Hallmark	Association with clinical characteristics and outcome	Ref.
circBMPR2	miR-553, USP4	N/A	MCF-7, MDA-MB-468, MDA-MB-231, T47D, HEK293T, ZR-75-1, SKBR3,	Tumour and adjacent normal tissues from 35 patients	Proliferation (-)	N/A	Liang et al. (2019a)
					Migration (-)		
					Invasion (-)		
					Apoptosis (+)		
circ-1073	HuR, C-Caspase 3/9, E-cadherin, Vimentin	Nude mice	MCF-10A, BT-549, MDA-MB-468, SK-BR-3, MDA-MB-231, T47D, MCF-7, ZR-75-1	Tumour and adjacent normal tissues from 112 patients	Proliferation (-)	Prognosis	Yi et al. (2020)
					Migration (-)	Tumor size	
					Invasion (-)	TNM stage	
					Apoptosis (+)		
					EMT (-)		
circ_0000442	miR-148b-3p, PTEN, PI3K-AKT pathway	Nude mice	MCF-10A, T47D, MDA-MB-231, MCF7, BT474, SUM-1315, SK-BR-3	Tumour and adjacent normal tissues	Proliferation (-)	Prognosis	Zhang and Mao (2021)
circCCDC85A	miR-550a-5p, MOB1A	Nude mice	BT-549, MDAMB-231, MCF-7	58 BC tissues and 40 normal breast tissues	Proliferation (-)	N/A	Meng et al. (2022)
					Migration (-)		
					Invasion (-)		
hsa_circ_0068033	miR-659	Nude mice	MCF10A, T47D, MCF-7, MDA-MB-468	Tumour and adjacent normal tissues from 36 patients	Proliferation (-)	Tumor size TNM stage	Yuan et al. (2020)
					Migration (-)		
					Invasion (-)		
					Apoptosis (+)		
hsa_circ_0072309	miR-492	Nude mice	T47D, MCF-7	32 tumor and adjacent normal tissues	Proliferation (-)	Prognosis	Yan et al. (2019)
					Migration (-)	Tumor size	
					Invasion (-)	Lymph node metastasis TNM stage	
circ-VRK1	N/A	N/A	MDA-MB-453, BT474, MCF7, MDA-MB-231	350 tumour and 163 adjacent normal tissues	Proliferation (-)	Tumor size	Li and Li (2020)
					Apoptosis (+)	Prognosis	
						TNM stage	
circNFIC	miR-658, UPK1A	Nude mice	MDA-MB-231, MDA-MB-468	Primary BC tissues and lung metastatic BC tissues, BC tissues from 150 patients	Proliferation (-)	Prognosis	Xu et al. (2020a)
					Migration (-)	Lymph node metastasis	
circTADA2As	miR-203a-3p, SOCS3	Nude mice	MDA-MB-231, MCF-7, MCF-10A	121 BC tissues and 16 normal mammary gland tissues, 57 TNBC samples	Proliferation (-)	Prognosis	Xu et al. (2019b)
					Migration (-)		
					Invasion (-)		
circ-ITCH	miR-214, miR-17, ITCH	Nude mice	MCF-10A, BT-549, T47D, MCF-7, MDA-MB-231, SK-BR-3	275 BC and 68 adjacent normal tissues	Proliferation (-)	Prognosis	Wang et al. (2019c)
					Migration (-)		
					Invasion (-)		
circRNA_000911	miR-449a, Notch1, NF-κB pathway	N/A	MCF-10A, MDA-MB-231, MCF-7, SKBR-3, MDA-MB-468, T47D, MDA-MB-453	Tumour and adjacent normal tissues from 35 patients	Proliferation (-)	Prognosis	Wang et al. (2018a)
					Migration (-)		
					Invasion (-)		
					Apoptosis (+)		
circRNA_103,809	miR-532-3p	N/A	MCF-10A, BT20, MDA-MB-157, MCF7, MDA-MB-468, MDA-MB-231, T47D	Tumour and adjacent normal tissues from 65 patients	Proliferation (-)	Distant metastasis	Liu et al. (2020b)
					Migration (-)	Tumor size	
					Invasion (-)	TNM stage HER-2 status	
					EMT (-)	Prognosis	
circFBXW7	miR-197-3p, FBXW7, FBXW7-185aa protien	Nude mice	MCF-10A, T47D, HCC38, MCF-7, BT474, MDA-MB-453, MDA-MB-231, MDA-MB-468, MA-891, BT549, 4T1, SKBR-3	Tumour and adjacent normal tissues from 473 patients	Proliferation (-)	Prognosis	Ye et al. (2019)
					Migration (-)	Tumour size Lymph node metastasis	
						TNM stage	
circ-LARP4	miR-424, miR-761	N/A	HMEC, MDA-MB-468, MCF-7, BT474, MDA-MB-231,	Tumour and adjacent normal tissues from 283 patients	Proliferation (-)	Tumor size Clinical stage Prognosis	Zhang et al. (2020)
					Migration (-)		
					Invasion (-)		
circAHNAK1	miR-421, RASA1	Nude mice	MCF10A, BT483, BT474, MCF-7, T47D, HCC1569, SKBR3, HCC 1806, BT549, HCC38, MDA-MB-453, MDA-MB-436, MDA-MB-468, MDA-MB-231	Tumour and adjacent normal tissues from 136 patients	Proliferation (-)	Prognosis	Xiao et al. (2019)
					Migration (-)	Tumor size	
					Invasion (-)	Lymph node metastasis	
						TNM stage	
circRNA_0001283	miR-187, HIPK3	Nude mice	MCF-10A, MDA-MB-231MCF-7, MDA-MB-453, MDA-MB-468	10 samples of Tumour and adjacent normal tissues	Proliferation (-)	N/A	Hu Y. et al. (2020)
					Migration (-)		
					Invasion (-)		
					Apoptosis (+)		
circASS1	miR-4443, ASS1	N/A	MDA-MB-231, MCF-7	N/A	Migration (-)	N/A	Hou et al. (2019)
					Invasion (-)		
circEHMT1	miR-1233-3p, KLF4	Nude mice	HMEpC, SK-BR-3, ZR-75-1, MCF-7, MDA-MB-231, MB-468, BT-549, T47D	Tumour and surrounding normal tissues from 42 patients	Migration (-)	Tumour size Prognosis	Lu et al. (2020)
					Invasion (-)	T’NM stage	
						Lymph node metastasis	
circNR3C2	miR-513a-3p, HRD1, vimentin	Mice	MCF-7, BT549, T-47D, BT474, HEK293, MDA-MB-231	70 tissues samples of BC	Proliferation (-)	Lymph node metastasis Prognosis	Fan et al. (2021)
					Migration (-)		
					Invasion (-)		
					EMT (-)		
circRNA_000554	miR-182, ZFP36	Nude mice	MCF-10A, MDA-MB-157, MDA-MB-231, SK-BR-3, SUM-159, MCF-7	138 tumour and 38 adjacent normal tissues from BC patients	Invasion (-)	Prognosis	Mao et al. (2020)
					Migration (-)		
					EMT (-)		
					Apoptosis (+)		
circDDX17	miR-605, CDK1, p21	N/A	HBL-100, MCF-7, BT549, HCC2218, MCF-10A, BT474	Tumour and adjacent normal tissues from BC patients	Proliferation (-)	Lymph node metastasis	Peng and Wen (2020)
					Apoptosis (+)	Tumour grade	
						Prognosis TNM stage	

Furthermore, Li et al. compared the expression of circ-VRK1 in 350 BC tumor tissues and 163 adjacent tissues, and the results showed lower circ-VRK1 expression in tumor tissues. They also found that patients with a low level of circVRK1 expression had unfavorable clinicopathological features (Li and Li, 2020). Likewise, Liu and his colleagues used plasmid transfection to overexpress circRNA_103809 in cell lines, results showed that overexpression of circRNA_103809 could significantly suppress the growth of these cell lines. The team also studied proteins that are involved in the cell cycle (CyB1 and Cyd1) and found that overexpression of circRNA_103809 could stop the cell cycle at the G2/M phase (Liu et al., 2020b). Additionally, Xiao et al. showed a low level of circAHNAK1 in TNBC patients and cell lines. Then, they overexpressed circAHNAK1 in BC cells and found that it inhibited cancer proliferation and suppressed colony formation in two different cell lines by affecting miR-421 and RASA1 (Xiao et al., 2019).

These findings shed light on previously unknown functions of circRNAs in the BC tumor microenvironment, particularly in tumor growth regulation.

### 4.2 Regulation of EMT and tumor metastasis

Breast cancer is a systemic disease that commonly leads to metastatic spread (Hüsemann et al., 2008). Metastasis is a complex and multi-step process (Lambert et al., 2017) that is responsible for the majority of cancer patients’ deaths (Wittekind and Neid, 2005). Therefore, knowing the mechanisms behind metastasis can be crucial to improve alternative therapies and creating new approaches for better managing metastatic diseases (Parker et al., 2022).

**FIGURE 3:**
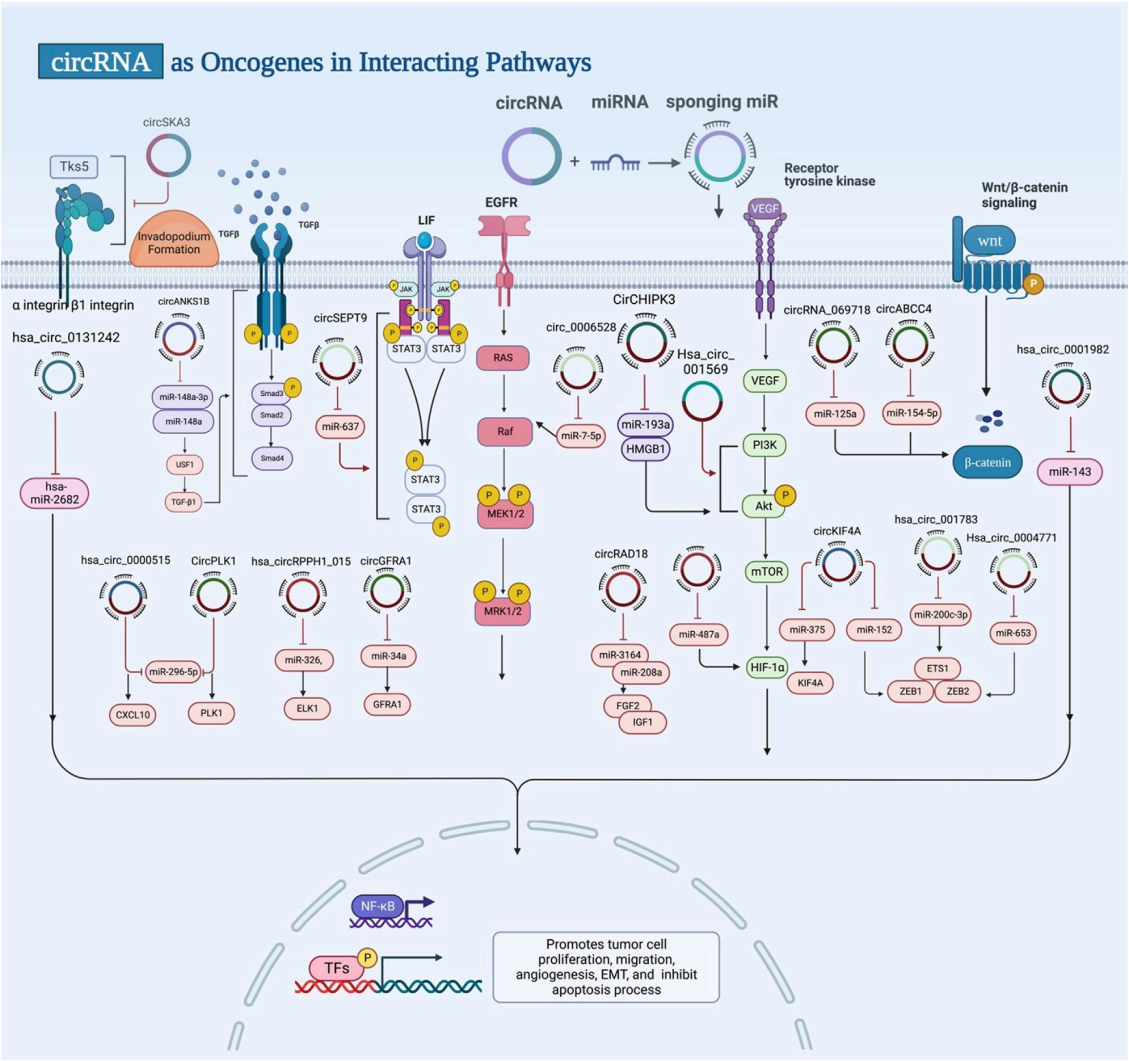
Exosomal circRNAs act as oncogenes. Exosomal circRNAs can promote tumor cell proliferation, migration, angiogenesis, EMT and inhibit apoptosis by targeting miRNA, genes, and signalling pathways.

Tumor exo-circRNAs are more highly expressed in BC (De Palma et al., 2022). Their significance in cancer metastasis and EMT was demonstrated through sponging miRNAs and modifying tumor suppressor genes or specific signalling pathways (Figure 3). For instance, Zeng and his colleagues showed that patients with overexpressed circANKS1B had a higher risk of metastasis to lymph nodes and higher clinical stage, as *in vitro* and *in vivo* research outcomes demonstrate that circANKS1B enhanced metastasis and invasion by inducing epithelial-to-mesenchymal transition (EMT) through TGF-β1 signalling pathway (Zeng et al., 2018). Meanwhile, Chen et al. detected overexpression of FECR1 circRNA in BC cell lines and tissue samples. Circular RNA FECR1 made tumors more aggressive and spread to other parts of the body by controlling how genes methylated and demethylated their DNA. It could do this by interacting with the FLI1 promoter *in cis* to start TET1 demethylase and by turning down DNMT1 *in trans* (Chen N. et al., 2018). Zhou et al. selected overexpressed hsa_circ_0011946 among 152 differentially expressed circRNA in BC. Based on miR26a/b sponging, they hypothesized that hsa_circ_0011946 targeted replication factor RFC3. When hsa_circ_0011946 was knocked down, RFC3 mRNA and protein expression were silenced. Thus, migration and invasion abilities were inhibited (Zhou et al., 2018). Moreover, it was discovered by Song et al. that hsa_circ_0007534, when elevated, promoted BC cell proliferation and invasion *via* MUC19 to modify miR-593 production, whereas hsa_circ_0007534, when downregulated, lost its oncogene properties and increased apoptosis (Song and Xiao, 2018).

Fu and his colleagues showed that circBCBM1 encourages cancer proliferation and migration. *In vivo* studies with mice showed that circBCBM1 also helps growth of cells and spread to the brain. Their finding suggested that circBCBM1 sponges miR-125a and modulates BRD4 regulation, leading to changes in the MMP9 expression *via* the Sonic hedgehog (SHH) signalling pathway (Fu et al., 2021). Also, Zhou et al. looked at BC and lung tissues that had spread to other parts of the body, and they found that metastatic lung samples had the most circFBXL5 upregulation. They showed that the knockdown of circFBXL5 in mouse models diminished the growth and lung metastasis of tumors. Furthermore, they revealed that circFBXL5 modulated the expression of SRSF6 *via* sponging miR-660 (Zhou et al., 2020a). Similarly, Song et al. detected circHMCU upregulation in BC cell lines, which was linked to the fast proliferative process and metastasize to the lungs. Furthermore, they found that circHMCU could impact the G1 phase cell cycle checkpoint and epithelial-mesenchymal transition (EMT) pathway of BC cells, enhancing proliferation, migration, and invasion (Song et al., 2020).

**FIGURE 4:**
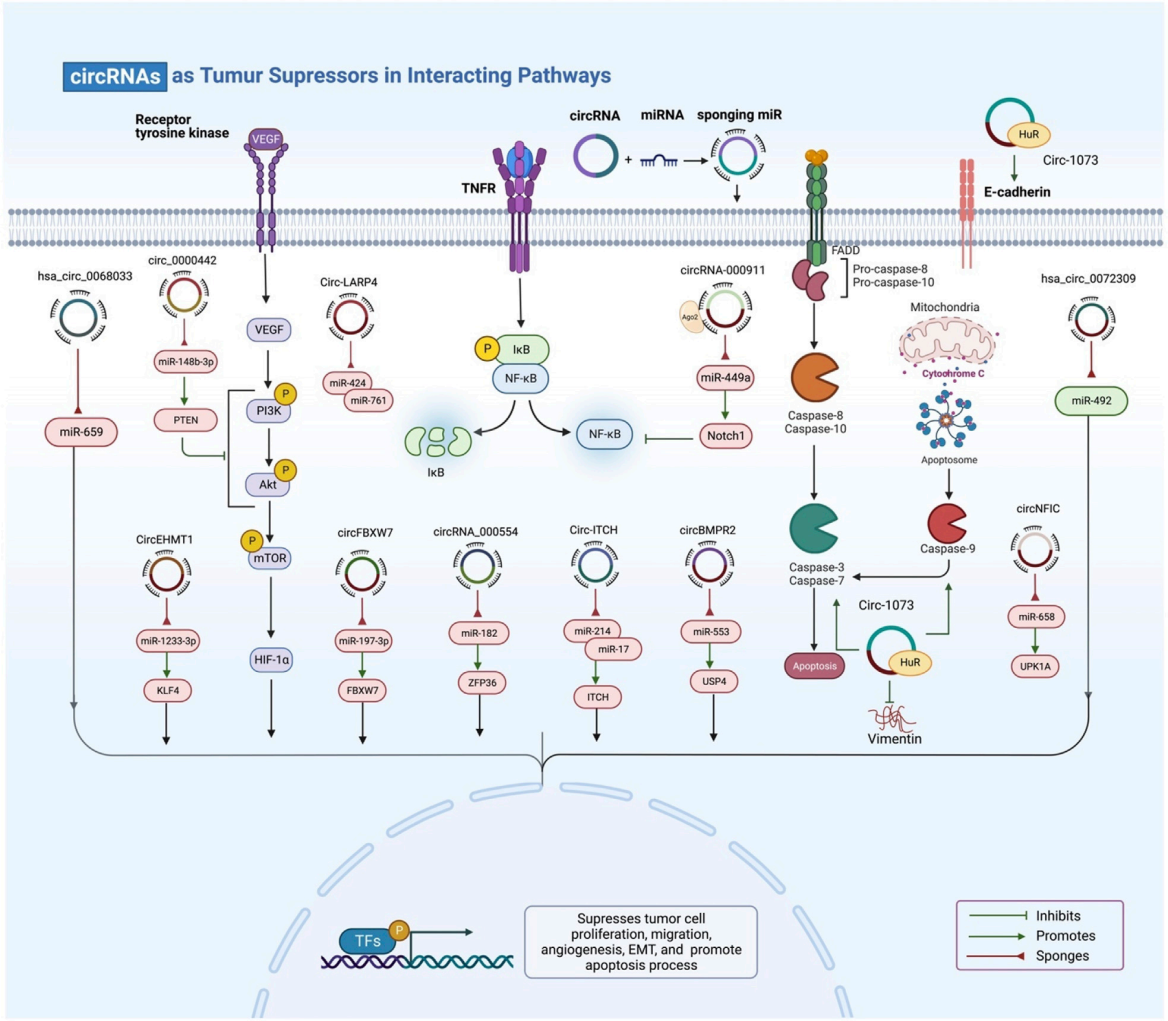
Exosomal circRNAs act as tumor suppressors. By targeting miRNA, genes, and a variety of signalling pathways, exosomal circRNAs can inhibit tumor cell proliferation, migration, angiogenesis, and EMT and stimulate apoptosis.

On the other hand, the expression levels of certain exo-circRNAs are reduced or inhibited in BC (Figure 4). For example, Xu and his team performed high-throughput circular RNA microarray assays of primary BC tissues and lung metastatic tissues samples and found circNFIC to be most downregulated in metastatic lung tissues among 20 dysregulated circRNAs. Then, they overexpressed circNFIC in mouse models and detected suppression of tumor growth and lung metastasis (Xu et al., 2020a). Hou and his team detected the most downregulation of circASS1 in 1,137 dysregulated circRNAs in BC cells. Overexpression of circASS1 was discovered to have anti-invasion and anti-migration effects. The group hypothesized that circASS1 promotes expression of its parent gene ASS1 *via* sponging miR-443 (Hou et al., 2019). Furthermore, significant downregulation of circNR3C2 in TNBC was discovered by Fan et al., and this was inversely linked with distant metastasis and tumor invasiveness. Overexpression of circNR3C2 inhibits tumor development and metastasis by degrading vimentin, as shown by gain-of-function studies. It also promotes expression of the tumor suppressor gene HRD1 (Fan et al., 2021). Moa et al. determined a low level of circRNA_000554 and ZFP36 while a high level of miR-182 in BC tissues. In addition, an immunofluorescence assay was performed to evaluate expressions of EMT markers Vimentin, N-cad, and E-cad in MCF-7 cells and the results showed reduced expression of Vimentin and N-cad, and increased expression of E-cad (Mao et al., 2020).

Additionally, circular mitochondrial RNAs (circ-mtRNAs) have been found to regulate mitochondrial function and cellular metabolism in BC. For example, a specific circ-mtRNA, called circRNA_103809, was significantly down-regulated in BC tissues compared to adjacent normal tissues (Liu et al., 2020b). In breast cancer cells, the overexpression of circRNA_103809 could interfere with the EMT signalling pathway, causing miR-532-3p to function improperly, resulting in G2/M phase arrest and a reduction of cell proliferation and metastasis (Liu et al., 2020b). However, further studies are required to better understand circ-mtRNAs’ functions and their potential as diagnostic or therapeutic targets in breast cancer.

These studies show that circRNAs can influence the spread of tumor cells to distant parts of the body through different mechanisms and pathways by suppressing or promoting invasion and metastasis.

### 4.3 Exosomal circRNAs and apoptosis

Apoptosis is programmed cell death that normally occurs through the developmental and aging stages of cells as a natural body mechanism to control the population of cells in tissues (Kaczanowski, 2016). Apoptosis occurs just in a single cell and does not trigger an immune response. The relative abundance of pro-apoptotic and anti-apoptotic signals within and around a cell determines whether the cell will survive or die by apoptosis (Henne et al., 2013).

**FIGURE 5:**
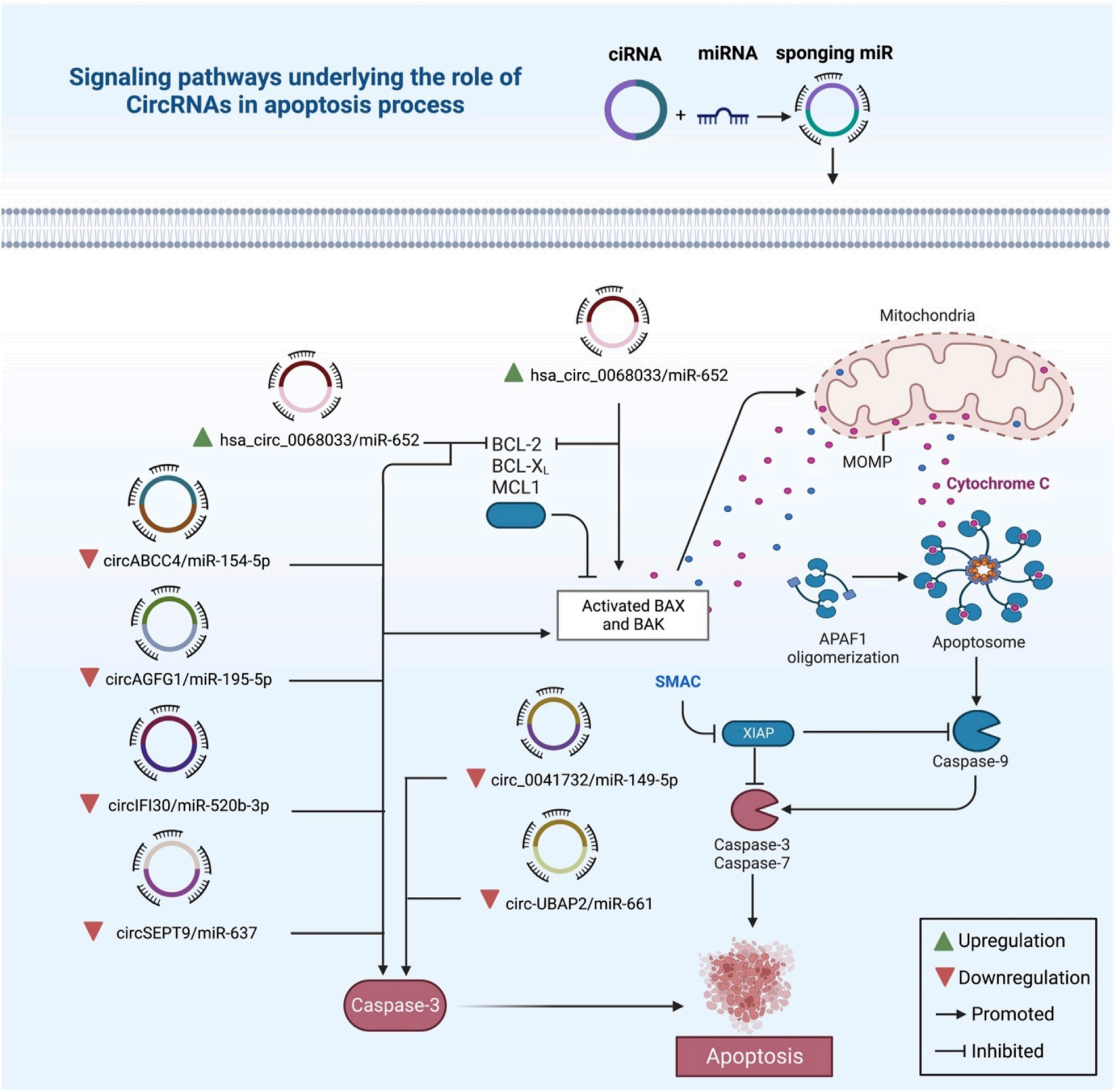
The intrinsic apoptotic pathway in breast cancer and the regulatory roles of exo-circRNAs. The production of caspase-3 is reduced when tumor suppressor circRNAs are downregulated, which in turn reduces apoptosis. Contrarily, oncogenic circRNAs like hsa_circ_0068033 promote oncogenic activity by inhibiting apoptosis *via* activating BAX and BAK genes.

Through interactions with downstream signalling pathways, circRNA can control the apoptotic process and contribute to BC pathogenesis (Figure 5). For example, Jiang and Cheng detected overexpression of circABCC4 in BC tissues, and they found circABCC4 to be involved in cell viability, migration and apoptosis *via* sponging miR-154-5p. Then, with the downregulation of circABCC4, they discovered enhancement of apoptosis, as well as significant downregulation of Bc1-2 and upregulation of Cleaved/Caspase-3 (Jiang and Cheng, 2020). Zheng et al. investigated the role of circSEPT9, and they found upregulation of circSEPT9 was associated with advanced clinical stage and bad prognosis in TNBC. They also revealed circSEPT9’s role in tumor growth, migration, invasion, and apoptosis *via* miR-637/LIF axis (Zheng et al., 2020). Likewise, Xie et al. detected high expression of hsa_circ_0004771 and ZEB2 in BC tumor tissues, with a reduction in the expression of miR-653. Differential knockdown of circ_0004771 and ZEB2, as well as overexpression of miR-653 in BC cells, decreased cell growth and triggered apoptosis (Xie et al., 2019).

PI3K/AKT signalling pathway is one of the main signal transition pathways inside cells, and they are remarkably important in apoptosis and cell survival regulation (Meng et al., 2017). According to recent evidence, the expression of cancer-related genes modulated by the circRNA/PI3K/AKT axis and suppressed apoptosis. For instance, Xu et al. detected significant upregulation of hsa_circ_001569 both in cell lines and BC tissues. When they glanced at the effects of silencing has_circ_001569 in cell lines, they found that has_circ_001569 knockdown not only stopped migration and invasion but also changed EMT markers and caused apoptosis by preventing the activation of the PI3K/AKT signalling pathway (Xu et al., 2019a).

On the other hand, the upregulation of circRNAs may induce the apoptosis process through sponging miRNAs. For example, Hu et al. found that the upregulation of circRNA_0001283 significantly inhibited cell growth and invasion while promoting apoptosis by sponging miR-187 and decreasing its expression (Hu Y. et al., 2020). Additionally, Yuan et al. detected downregulated hsa_circ_0068033 in BC tissues, which was significantly associated with the TNM stage and tumor size of patients. Furthermore, Yuan and his colleagues performed *in vitro* experiments and also found overexpressed hsa_circ_0068033 to sponge miR-659 and inhibit tumor growth in addition to inducing apoptosis through activation of intrinsic apoptotic pathways (Yuan et al., 2020). Furthermore, Wang et al. detected circRNA_000911 to be downregulated in BC cells. When they overexpressed circRNA_000911 in cell lines, they observed suppression of proliferation, migration, and invasion, with increased apoptotic abilities of the cells through sponging miR-449a, thus enhancing Notch1 and NF-κB signalling pathway (Wang et al., 2018a). Focusing on mechanisms in which circRNAs affect apoptosis is essential in BC since studies show that treating BC through promoting apoptosis is a promising approach.

### 4.4 Modulation of chemotherapy resistance

Therapy for breast cancer consists of multifaceted strategies, including surgery, radiotherapy, neoadjuvant and adjuvant therapy. It is crucial that the chosen therapy has an optimal therapeutic effect for the proper treatment of BC (Fisusi and Akala, 2019).

Tamoxifen is known as an endocrine therapy that is most frequently used for hormone receptor-positive (HR positive) patients suffering from BC. Sang and his team used RNA-seq to detect the downregulation of circRNA_0025202 in tamoxifen-resistant cells, they performed further analysis and found that circRNA_0025202 acted as a tumor suppressor. Then they treated BC cells with circRNA_0025202 alongside tamoxifen, which led to tumor growth suppression and enhanced tamoxifen sensitivity *in vivo* (Sang et al., 2019). Similarly, Liang et al. detected downregulation of circBMPR2 in BC tissues. They knocked down circBMPR2 in tamoxifen-treated cell lines, and assay results showed enhanced tamoxifen resistance by suppressing tamoxifen influenced apoptosis (Liang et al., 2019a).

On the other hand, Hu et al. found that circ_UBE2D2 was upregulated in BC tamoxifen-resistant cells and promoted resistance through combination with miR-200a-3p to modulate viability of cells, metastasis, and Erα level *in vivo* and *in vitro* studies (Hu K. et al., 2020).

Monastrol is a chemotherapeutic agent that suppresses tumors by hindering mitotic kinesin Eg5 needed for forming bipolar spindle. Liu et al. performed a human circRNA microarray on cancer cells resistant to monastrol and found circRNA-MTO1 to be downregulated. The team then upgraded circRNA-MTO1 expression in BC cells resistant to monastrol and showed that circRNA-MTO1 not only reversed resistance but also had a synergetic effect when used with monastrol through binding to TRAF4 and inhibiting Eg5 protein (Liu et al., 2018). Even though BC cells showed over-expression of circFBXL5, even more upregulation was detected in antitumor agent 5-fluorouracil (5-FU) resistant BC cells. According to study results performed by Zhu and his colleagues, circFBXL5 appeared to regulate cell migration, invasion, and apoptosis by modulating the miR-216b/HMGA2 axis (Zhu et al., 2021).

In contrast, Liang et al. detected circKDM4C downregulation in metastasized tissues of BC, which was involved in both BC advancement and chemotherapy resistance. Resistant cells to doxorubicin showed a low level of circKDM4C, while transfection of MDA-MB-231/DOX cells with circKDM4C expression vector led to reduced cell proliferation and lowered resistance (Liang et al., 2019b). Likewise, Zhang et al. found downregulation of circKDM4C in BC tissues and intended overexpression of circKDM4C increased sensitivity of doxorubicin-resistant cells (Zhang et al., 2020). Another circular RNA that impacted therapy with doxorubicin was circUBE2D2, but negatively, as it induced doxorubicin resistance. When Dou and his colleagues knocked down circUBE2D2 in TNBC cells, it reversed the chemoresistance *via* downregulation of miR-512-3p or upregulation of CDCA3 (Dou et al., 2020). Similarly, overexpression of hsa_circ_0092276 was detected in BC doxorubicin-resistant cells by Wang et al., and this overexpression contributed to the resistance through altering autophagy-related gene 7 (ATG7) *via* sponging miR-384 (Wang et al., 2021).

Resistance to paclitaxel is one of the concerns in TNBC patients undergoing chemotherapy. Ma and his colleagues overexpressed circAMOTL1 in BC cells through plasmid construct, which improved cell viability, reduced apoptosis, and promoted invasion ability in MDA-MB-231 cells treated with paclitaxel. CircAMOTL contributed to resistance *via* AKT pathway regulation, promotion of anti-apoptotic protein, and suppression of pro-apoptotic protein (Ma et al., 2019). Meanwhile, Zang et al. observed circ-RNF111 upregulation in cells resistant to paclitaxel compared to cells responding to paclitaxel. When the team knocked down Circ-RNF111, the resistance of BC cells to paclitaxel was suppressed both *in vivo* and *in vitro* by upregulating E2F3 *via* sponging miR-140-5p (Zang et al., 2020). Li et al. detected 3.34 times increase of hsa_circ_0000199 in TNBC tissues compared to non-TNBC tissues. Breast cancer cells with silenced hsa_circ_0000199 became more sensitive to paclitaxel, cisplatin, gemcitabine, and adriamycin therapies in TNBC. Improved chemotherapeutic outcomes were achieved through enhanced expression of miR-206/miR-613 and inactivated PI3K/Akt/mTOR signalling (Li et al., 2021). In addition, Goa and his colleagues spotted a higher level of circ_0006528 in BC cells resistant to adriamycin compared to sensitive cells, and even though the mechanism was not known, there was a noticeable increase in sensitivity to adriamycin with downregulation of circ0006528 (Gao et al., 2017) (Table 3).

**TABLE 3:** Exo-circRNAs in breast cancer promotes therapeutic resistance.

Therapeutic resistance	Types of Circular RNA	Regulation	Effect	Targeted pathway/Axis	Ref.
5-Fluorouracil	circFBXL5	Upregulated	Promote resistance	miR-216b/HMGA2	Zhu et al. (2021)
Doxorubicin	circKDM4C	Downregulated	Promote sensitivity	miR-548p/PBLD	Liang et al. (2019b)
	circUBE2D2	Upregulated	Promote resistance	miR-512-3p/CDCA3	Dou et al. (2020)
	circ-LARP4	Downregulated	Promote sensitivity	miR-424	Zhang et al. (2020)
	hsa_circ_0092276	Upregulated	Promote resistance	miR-348/ATG7	Wang et al. (2021)
Tamoxifen	circRNA_0025202	Downregulated	Promote sensitivity	miR-182-5p/FOXO3	Sang et al. (2019)
	circ_UBE2D2	Upregulated	Promote resistance	miR-200a-3p	Hu K. et al. (2020)
	circBMPR2	Downregulated	Promote sensitivity	miR-553/USP4	Liang et al. (2019a)
Paclitaxel	circ-RNF111	Upregulated	Promote resistance	miR-140-5p/E2F3	Zang et al. (2020)
	circAMOTL1	Upregulated	Promote resistance	AKT pathway	Ma et al. (2019)
	circGFRA1	Upregulated	Promoted resistance	miR-361-5p/TLR4	Zheng et al. (2021)
	circ-ABCB10	Upregulated	Promoted resistance	let-7a-5p/DUSP7	Yang et al. (2020)
Lapatinib	circ-MMP11	Upregulated	Promote resistance	miR-153-3p/ANLN	Wu et al. (2021)
Monastrol	circRNA-MTO1	Downregulated	Promote sensitivity	TRAF4/Eg5 axis	Liu et al. (2018)
Adriamycin	circ_0006528	Upregulated	Promote resistance	N/A	Gao et al. (2019)
	circ_0001667	Upregulated	Promoted resistance	miR-4458/NCOA3	Cui et al. (2022)
	circ_0085495	Upregulated	Promote resistance	miR-873-5p/integrin β1	Xie and Zheng (2022)

It can be observed through these studies that circRNAs have essential roles in the efficacy of a wide variety of treatment options for BC through their contribution to resistance or sensitivity of therapies.

## 5 Role of exosomal circRNAs in diagnosis and prognosis

Exosomes contain many biologically active agents with the potential to be used as biomarkers in BC (Jia et al., 2017), including circRNAs (Jahani et al., 2020). Two characteristics of circRNAs that make them a suitable candidate for biomarkers of BC are: they are remarkedly stable in comparison to linear RNAs, and they have ten times more expression than linear isomers (Guo et al., 2014). Furthermore, there is evidence suggesting that circRNAs have a crucial part in BC progression and can be used for BC screening as tumor markers due to being stable and tissue-specific (Lü et al., 2017).

Determining biomarkers has a crucial role in diagnosing, anticipating prognosis, and identifying the clinical importance of BC patients. Liang et al. detected 1.5-fold overexpression of linear CDYL and 3.2-fold elevation of circular CDYL expression in BC tissues compared to paired noncancerous tissues. However, expression of circCDYL was associated significantly with clinicopathological characteristics of BC patients, while linear CDYL was not. Therefore, Liang and his colleagues suggest that circCDYL has the capacity to help in diagnosis and prognosis in BC (Liang et al., 2020). Furthermore, Yin et al. performed ROC curve to analyze the diagnostic value of hsa_circ_0001785, and their results revealed that hsa_circ_0001785 had 0.784 AUC value, supporting its great potential to be a biomarker for BC early detection. Similarly, Yuan et al. suggested that hsa_circ_0068033 is a valuable biomarker for early detection of BC for the reason that ROC analysis showed that hsa_circ_0068033 has a high diagnostic value (AUC = 0.8480) (Yuan et al., 2020).

Additionally, He et al. used. Kaplan-Meier survival analysis to examine prognostic determinant abilities of circGFRA1. According to results used to draw OS and DFS graphs, over-expressed circGFRA1 was linked to bad prognosis in TNBC patients (He et al., 2017). Furthermore, in two different studies, circEPSTI1 and circKIF4A were investigated in 240 triple-breast cancer patients; results showed overexpression of both circular RNAs. The high expression level of both was direct relation to size of the tumor, lymph node invasion, stage of TNM, as well as bad prognosis (Chen B. et al., 2018; Tang et al., 2019). Similarly, Qu et al. discovered that overexpressed circ_0103,552 in BC patients appeared to be correlation with bad pathological features and fewer survival chances. The independent prognostic factor of circ_0103,552 was also examined by multivariate analysis, and results showed circ_0103,552 to be an independent prognostic predictor for BC (*p* = 0.042) (Yang et al., 2019b). Additionally, Chen et al. detected overexpression of cirCHIPK3 in tumor tissues, which assisted BC cells in developing and progressing through modulating HMGB1/PI3K/AKT, and its high level was associated closely with lower prognosis (Chen et al., 2020).

On the contrary, Yin and his colleagues measured hsa_circ_0001785 plasma levels in BC patients before and after surgery, and it showed a significant decrease in hsa_circ_0001785 in postoperative patients (Yin et al., 2018). Likewise, Zhang et al. performed RT-qPCR on sample tissues of 283 female BC patients, and the level of circ-LARP4 appeared to be low. Downregulated circ-LARP4 at the tumor site of BC was related to increased tumor size, advanced clinical stage, and bad prognosis in BC (Zhang et al., 2020). Furthermore, Liang et al. detected downregulation of circKDM4C in BC cells through microarray results, and the low level was linked to bad prognosis, metastasis, and low survival chances (Liang et al., 2019b).

Being an effective indicator of both diagnosis and prognosis, circRNAs have the potential to be used to detect early tumor formation and to predict the survival chance of BC patients.

## 6 Exosomal circRNAs as potential therapeutic targets in breast cancer

Breast cancer is very heterogenous, and despite advancements in therapies, the prognosis of the disease is still not very promising. Thus, novel and specific mechanism-based drugs should be designed and synthesized for treating BC. Recent studies related to circRNA in cancer showed that circRNA could be used as a therapeutic strategy (Hussen et al., 2022; Wu et al., 2022).

Ye and his colleagues detected downregulation of CircFBXW7 in BC cell lines, and the expression level was associated with tumor size and metastasis of patients. When circFBXW7 was overexpressed in cell lines using a vector, it sponged miR-197-3p, increasing tumor suppressor gene FBXW7 expression, which encoded for FBXW7-185aa protein. Consecutively, the migration and proliferation capabilities of cells were diminished, suggesting circFBXW7 to be a good target for new therapies and a prognostic biomarker of BC (Ye et al., 2019). In another most recent study supporting the use of circRNA as a therapeutic target for TNBC, Li et al. detected upregulation of circ_0041732 in cancer cells, which contributed to tumor formation *via* sponging miR-149-5p. Fibroblast growth factor 5 (FGF5) is considered a target for miR-149-5p. Silencing of circ_0041732 lead to tumor formation inhibition by reducing FGF5 expression *via* miR-149-5p *in vivo* (Li et al., 2022a). Liang et al. spotted a low expression level of circBMPR2 in BC cells, that was negatively linked to cell proliferation, invasion, and migration in BC. According to the recent data, circBMPR2 to profusely sponge miR-553, and miR-553 promoted cancer cells to proliferate and migrate through tumor suppressor USP4. Thus, circBMPR2/miR-553/USP4 could be an excellent therapeutic candidate (Liang et al., 2019a).

Similarly, Liu et al. detected downregulation of circRNA_103,809 in BC tissues, which was linked to tumor size, stage of TNM, and HER-2 levels. While intended overexpression of circRNA_103809 was able to suppress EMT pathway substantially *via* sponging miR-532-3p (Liu et al., 2020b). On the contrary, Huang and his colleagues detected upregulation of circ_0103,552, which acted like an oncogene by overexpressing CYR61 through targeting miR-515-5p and consequently promoting division, migration, and invasion characteristics. circ_0103552/miR-515-5p/CYR61 axis has a good aptitude to be developed for the treatment of BC (Huang et al., 2021).

Another therapeutic approach that can be developed from circRNA is pathway interactions. Wang et al. detected considerable downregulation of circRNA_000911 in BC cells, and through a biotin-labelled circRNA_000911 probe, they found that circRNA_000911 sponges miR-449a, consecutively modulating signalling flow of NF-κB and Notch1 (Wang et al., 2018a). Further, Chen et al. detected upregulation of cirCHIPK3 in BC tissues, which increased the expression of HMGB1 when sponging miR-193a (Chen et al., 2020). PI3K/AKT axis has an essential role in tumor progression (Chen et al., 2020), and HMGB1 has been shown to encourage angiogenesis and migration of tumors through PI3K/AKT signalling. Likewise, Western blot analysis revealed that HMGB1-mediated phosphorylation of PI3k and AKT was diminished in cells with silenced cirCHIPK3. Thus, suggesting that cirCHIPK3 is a very valuable candidate for finding novel therapies for BC (Chen et al., 2020).

Over the last few decades, a great deal of attention has been given to the development and evaluation of nanoparticles based on their potential to be utilized as good agents for cancer diagnosis and therapy (Baetke et al., 2015), with BC included (Mu et al., 2017). For example, When Yi et al. injected a mouse tumor site with nanoparticles containing circ-1073 plasmid, they concluded that the circ-1073 could bind to HuR to increase its expression, which in turn suppressed malignancy of BC cells by elevating E-cadherin and cleaved-Caspase 3/9 levels (Yi et al., 2020).

In particular, using exo-circRNAs as a way to treat cancer has both challenges and potential opportunities, especially when it comes to regulating circRNA expression *in vivo*, off-target effects, circRNA delivery, and drug resistance. Many approaches and strategies have been proposed to overcome these challenges.

In term of circRNAs regulating circRNA expression *in vivo*, Researchers proposed to use transposon to deliver a circRNA expression cassette (Mecozzi et al., 2022). In addition, it is possible to produce circRNAs artificially and enhance their capabilities (Rossbach, 2019).

On the other hand, the off-target limitation affects the capacity of circRNAs in BC therapy. To overcome this challenge siRNA approach can be used to reduce the expression of none-desired circRNA which may decrease the off-target effects of circRNA. For example, Zhang and his colleagues used siRNA to reduce the expression of none-desired circRNA which consequently decreased the off-target effects of circRNA (Zhang et al., 2018a).

Besides that, delivery of circRNA using lipid nanoparticles and delivery of circRNA *via* exosomes can be used to overcome delivery challenges. For example, according to genome-wide RNA-seq research, Li et al. found that exosomes are a rich source of circRNAs, and they were proven to be abundant in exosomes as compared to parental cells (Lener et al., 2015). Similarly, in a mouse model, Li and his team showed that circRNA can be successfully encapsulated and distributed into aggressive tumors by using lipid nanoparticles (Li et al., 2022b).

Additionally, the development of resistance to anti-cancer drugs is another issue that complicates BC therapy. Researchers found that tumor cells might use exosomes to handle their drug resistance *via* ciRS-122 to susceptible cells (Xu et al., 2020b).

## 7 Conclusion and future direction

Exosomes are nanometric particles that mediate the transfer of local or systemic cell-to-cell oncogenic signals and play a crucial role in driving cancer progression by providing a suitable environment for cancer cell proliferation and invasion. Exo-circular RNAs can act as oncogenes or tumor suppressors in cancer. They are involved in hallmarks of BC such as, proliferation, angiogenesis, migration, invasion, metastasis, and apoptosis through modulating different pathways. Breast cancer therapy can also be affected by exo-circRNAs, as they can contribute to the promotion of sensitivity and resistance to therapeutic drugs. Therefore, exo-circRNAs have the potential to be used as biomarkers or prognostic markers. In the future direction, practical treatment approaches can be developed from circRNA as they contribute to the formation and progression of BC through many different mechanisms and pathways. Especially due to its potential clinical value, studying circRNAs as cancer therapies is an exciting but difficult and challenging field of study. Advances in the research have shown their therapeutic value, and their prospective significance in individualized diagnostics and therapy. Nevertheless, their potential value as clinical studies for BC have not been evaluated in large clinical cohorts like that of other ncRNAs. The lack of methodological standards and the troubling methodological heterogeneity are two issues that, in our opinion, ought to be addressed in future studies. Large and more characterized cohorts, adequate validated procedures, proper control groups, and understanding BC tissue heterogeneity are required for applying into clinical translation. Accordingly, more research is needed to fully realize the value of exo-circRNAs and integrate them into the design and synthesis of novel therapies for BC.

## Glossary

ANLN: Anillin, Actin Binding Protein
ASS1: Argininosuccinate Synthase 1
ATG7: Autophagy Related 7
CDCA3: cell division cycle associated 3
CDK: Cyclin-Dependent Kinase
CircRNA: circular RNA
CYR61: Cysteine-rich angiogenic inducer 61
E2F3: E2F Transcription Factor 3
Exo-circRNA: exosomal circularRNA
FFPE: formalin-fixed, paraffin embedded
FGF2: Fibroblast Growth Factor 2
FOXO3: Forkhead Box O3
HIF-1α: hypoxia-inducible factor-1α
HIPK3: Homeodomain Interacting Protein Kinase 3
HMGA2: high-mobility group AT-hook 2
HRD1: E3 ubiquitin-protein ligase HRD1
IGF1: Insulin Like Growth Factor 1
MMP9: matrix metallopeptidase 9
MVEs: multivesicular endosomes
MTA1: Metastasis Associated 1
NCOA3: Nuclear Receptor Coactivator 3
NF-κB: Nuclear factor-κB
Notch1: Neurogenic locus notch homolog protein 1
p53: Tumor Protein P53
PAK1: p21 protein-activated kinase 1
PTEN: Phosphatase and Tensin Homolog
RASA1: Ras p21 protein activator 1
RFC3: Replication factor C subunit 3
SOCS3: Suppressor of Cytokine Signalling 3
SRSF6: Serine and Arginine Rich Splicing Factor 6
TLR4: Toll Like Receptor 4
TNBC: Triple negative breast cancer
TNM: Tumor (T), nodes (N), and metastases (M)
TRAF4: TNF Receptor Associated Factor 4
